# EUS-guided ablation of a symptomatic renal cyst in a patient with autosomal dominant polycystic kidney disease

**DOI:** 10.1097/eus.0000000000000153

**Published:** 2025-12-15

**Authors:** Jessica Arnold, Sebastian Zundler

**Affiliations:** Department of Medicine 1, University Hospital Erlangen, Erlangen, Germany.

A 66-year-old Caucasian woman with a history of autosomal dominant polycystic kidney disease (ADPKD) was referred to our center for EUS-guided drainage of a large pancreatic pseudocyst as diagnosed by magnetic resonance imaging (MRI) [Figure 1] and abdominal ultrasound [Figure 2]. She presented in reduced general condition and reported abdominal upper left quadrant pain and nausea for over 6 months. Laboratory results showed no signs of inflammation, and lipase levels were normal. Serum creatinine was mildly elevated, and the patient had mild microcytic hypochromic anemia.

**Figure 1:**
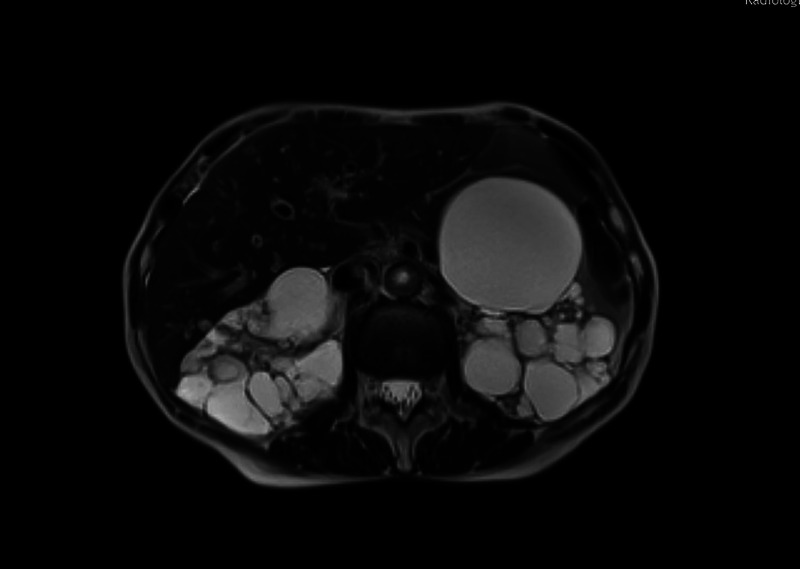
Initial magnetic resonance imaging demonstrating a large cystic lesion adjacent to the pancreas along with polycystic kidney disease.

**Figure 2:**
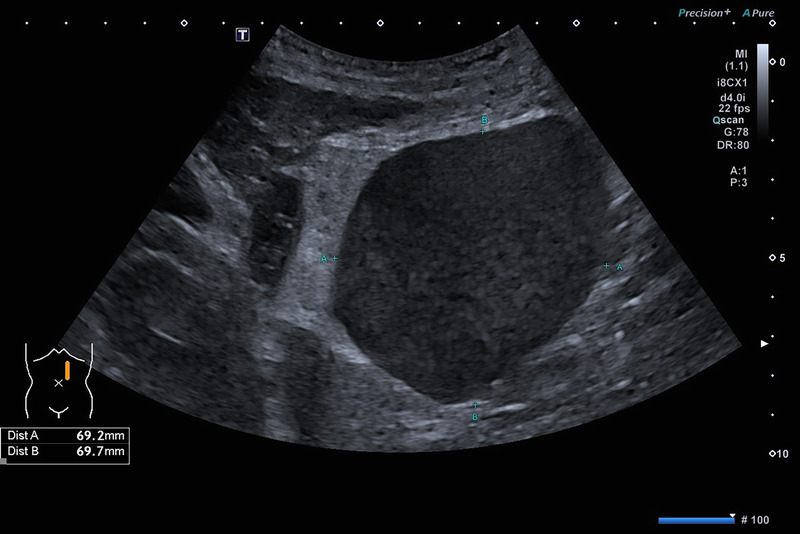
Abdominal ultrasound of the cystic lesion.

EUS showed a cyst with 8-cm diameter between the pancreatic tail, spleen, and left kidney [Figure 3]. Due to its thin-walled appearance, completely anechoic texture, and the direct relation to the polycystic kidney as well as the missing history of pancreatitis, we diagnosed a symptomatic renal cyst rather than a pancreatic pseudocyst.

**Figure 3:**
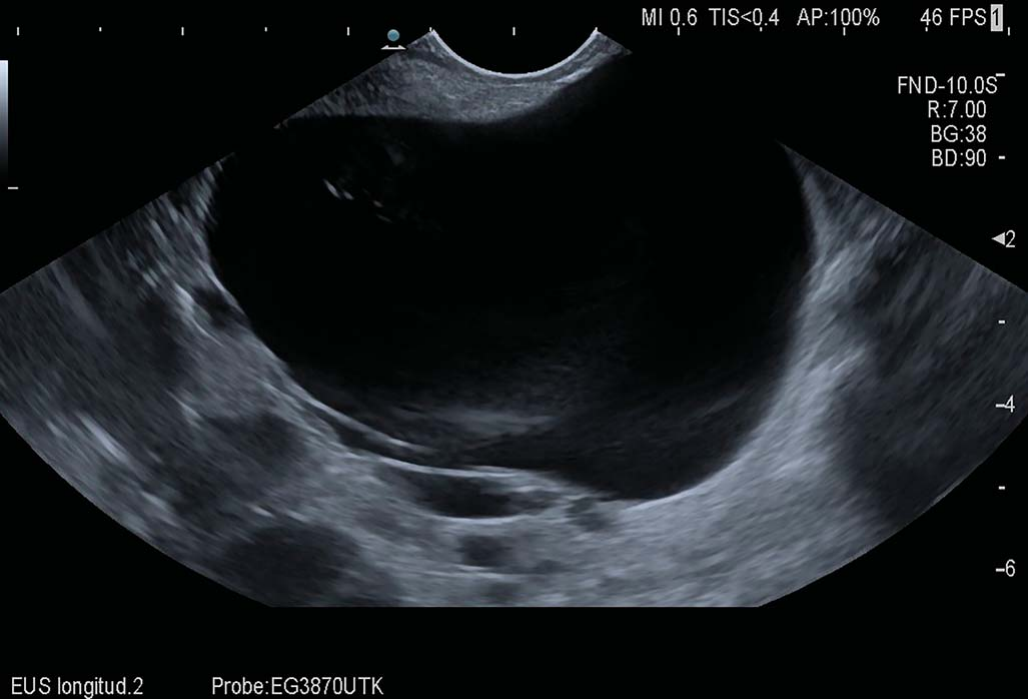
Appearance of the cystic lesion on EUS.

Because the patient was not considered a good candidate for surgery, we decided to perform EUS-guided ethanol ablation of the cyst. Thus, puncture with a 19G fine-needle aspiration (FNA) needle was performed, and 160 mL of clear, slightly yellowish fluid was aspirated [Figure 4]. Subsequently, 80 mL of 95% ethanol was instilled, left within the cyst for 10 minutes and subsequently re-aspirated.

**Figure 4:**
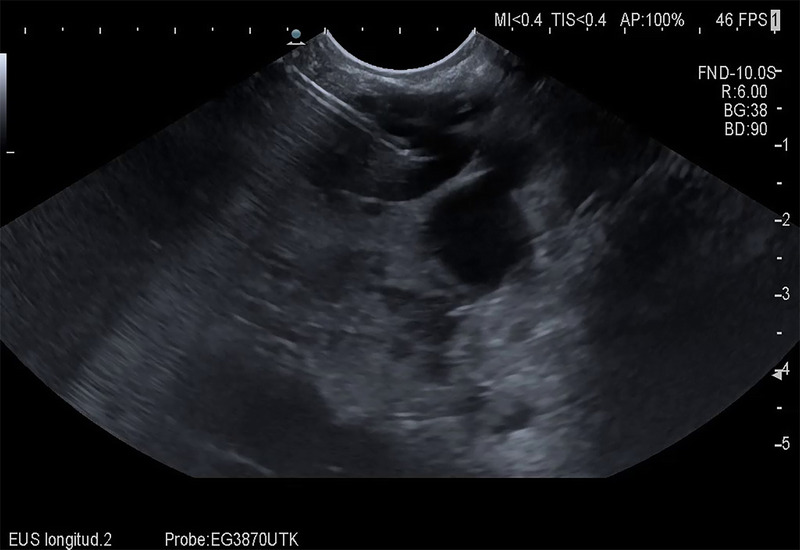
Image obtained during aspiration of cyst fluid after EUS-guided puncture of the cyst with a 19 FNA needle.

No immediate complications occurred, systemic ethanol levels remained negative, and the abdominal pain and nausea fully resolved. On follow-up abdominal ultrasound 3 days later, the cyst was no longer visible [Figure 5]. Similarly, 4 weeks later, MRI confirmed a complete regression of the cyst [Figure 6].

**Figure 5:**
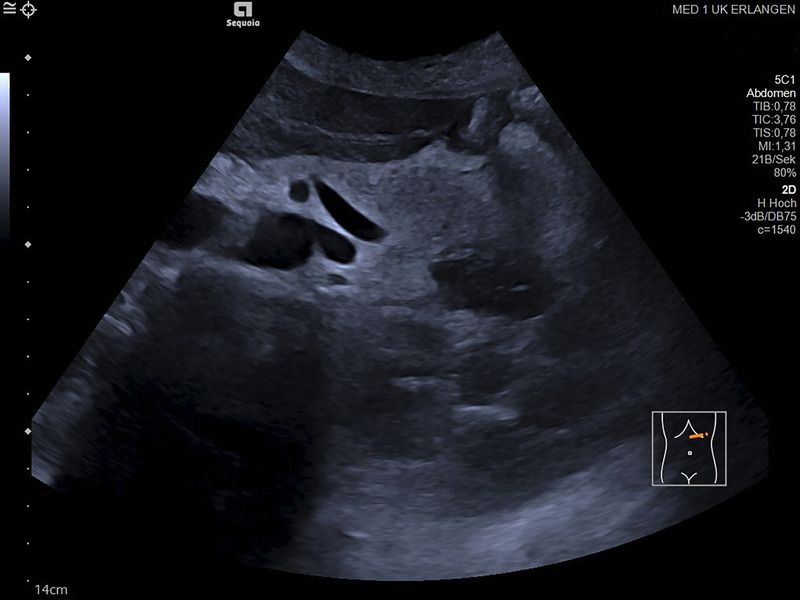
Abdominal ultrasound 3 days post-intervention.

**Figure 6:**
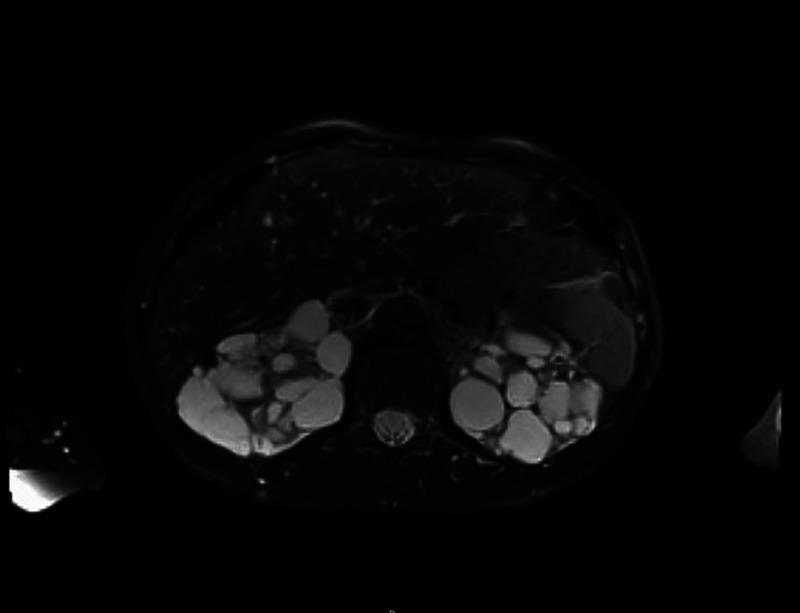
Magnetic resonance imaging 4 weeks post-intervention demonstrating complete resolution of the cyst.

This is one of the first reports of EUS-guided ethanol ablation of a renal cyst. Previously, percutaneous ethanol ablation of hepatic and renal cysts has been described to be safe and effective.^[1–3]^ Consistently, in our patient, this approach achieved full resolution of the cyst along with immediate symptom relief. Thus, endotherapy of symptomatic renal cysts may be considered on a case-by-case basis depending on accessibility and fitness for surgery.

## Ethical Approval

Not applicable.

## Informed Consent

Patient consent for publication was obtained.

## Source of Funding

None.

## Conflicts of Interest

None

## Author Contributions

Both authors contributed to the study conception and design. Intervention was performed by Sebastian Zundler, follow-up care was co-ordinated by Jessica Arnold. The authors jointly wrote the manuscript and approved the final version.

## Data Availability Statement

None
